# Clinical Challenges in Triple-Negative Breast Cancer (TNBC): Current Treatment Options, Their Limitations, and Emerging Perspectives

**DOI:** 10.3390/cancers18203403

**Published:** 2026-10-21

**Authors:** Rubismita Deka, Thiyam Ramsing Singh

**Affiliations:** Department of Molecular Biology and Biotechnology, Tezpur University, Napaam, Tezpur 784028, Assam, India

**Keywords:** breast cancer, TNBC, molecular subtypes, recurrence, drug resistance

## Abstract

Triple-negative breast cancer (TNBC) is characterised by the absence of expression of oestrogen receptor (ER), progesterone receptor (PR), and human epidermal growth factor receptor 2 (HER2). Compared with other breast cancer subtypes, TNBC is generally associated with a more aggressive clinical course, in part because of its marked molecular heterogeneity. Consequently, the availability of effective targeted therapies remains limited. Chemotherapy continues to be a major component of treatment, although its clinical benefit may be constrained by toxicity and the development of drug resistance. This review examines the principal clinical challenges in TNBC, evaluates currently available therapeutic approaches and their limitations, and discusses important areas that remain insufficiently addressed. Particular emphasis is placed on recent advances in emerging treatment strategies. Together, these developments may improve our understanding of TNBC and support future research towards more precise and personalised therapeutic approaches.

## Simple Summary

Triple-negative breast cancer (TNBC) is an aggressive and clinically complex form of breast cancer that presents substantial challenges in diagnosis, treatment, and long-term management. This review discusses the major clinical challenges associated with TNBC, together with the currently available treatment options and their limitations. It also examines emerging therapeutic approaches, including biomarker-guided therapy, immunotherapy, combination strategies, and targeted protein degradation. By linking the principal clinical problems in TNBC with current and emerging treatment strategies, this review aims to clarify where existing therapies remain inadequate and how newer approaches may help address these limitations.

## 1. Introduction

Cancer is characterised by the uncontrolled proliferation of abnormal cells that can invade surrounding tissues and, at advanced stages, metastasise to distant organs through the bloodstream or lymphatic system [1]. According to the World Health Organization (WHO), cancers of the lung, liver, and stomach rank among the most prevalent malignancies worldwide and contribute substantially to cancer-related mortality. Lung cancer remains a leading cause of cancer-related death among men, whereas breast cancer is the most frequently diagnosed cancer in women and a major cause of cancer-related mortality [2]. Approximately 20.6 million new cancer cases and 9.8 million cancer-related deaths were reported in 2024. Lung cancer accounted for the largest proportion of new diagnoses (12.8%), followed by female breast (11.8%), colorectal (9.9%), prostate (7.5%), and stomach cancers (4.7%) [3].

Breast cancer (BC) arises from epithelial cells of the mammary ducts or lobules and comprises a heterogeneous group of malignancies that can progress to invasive carcinoma. Breast cancer can metastasise to regional lymph nodes and distant organs [4]. Although it affects both sexes, it is considerably more common in women [5]. Globally, breast cancer accounts for approximately one in four cancer diagnoses among women and remains a major cause of cancer-related death, with an estimated 694,000 deaths reported in the 2024 WHO cancer statistics. Breast cancer is classified into molecular subtypes according to the expression of hormone receptors and human epidermal growth factor receptor 2 (HER2), including hormone receptor-positive (HR+), HER2-positive, and TNBC [6]. These subtypes require distinct therapeutic approaches: HR+ tumours are generally treated with endocrine therapy, whereas HER2-positive tumours are treated with HER2-targeted therapies. By contrast, TNBC is commonly managed with chemotherapy, immunotherapy, and selected targeted therapies (National Cancer Institute, 2026). Thus, the biological behaviour, prognosis, and therapeutic response of breast cancer vary by subtype, underscoring the need for tailored treatment strategies.

Triple-negative breast cancer (TNBC) accounts for approximately 10–20% of breast cancer cases, although its prevalence varies among populations [7]. Although the precise aetiology of TNBC remains incompletely understood, its development is considered multifactorial, reflecting interactions among lifestyle, environmental, hormonal, and genetic factors [6,8]. Genetic predisposition, including pathogenic alterations in tumour-suppressor genes such as BRCA1, together with modifiable factors such as diet, physical activity, and exposure to environmental carcinogens, may contribute to TNBC pathogenesis [9]. Epidemiological studies have also identified reproductive and metabolic factors, including early menarche, younger age at first full-term pregnancy, and higher body mass index (BMI), as potential contributors to TNBC risk [6].

The pathophysiology of TNBC is characterised by extensive genomic instability and clonal variation, with recurrent alterations affecting genes and pathways involved in tumour suppression, DNA repair, cell growth, and survival [10,11]. Interactions between these molecular abnormalities, changes in the tumour microenvironment, and cellular plasticity promote tumour-cell proliferation, invasion, and disease progression [12]. This biological complexity contributes to the marked clinical heterogeneity of TNBC and complicates its therapeutic management [13].

Current therapeutic options for TNBC remain limited in efficacy, contributing to unfavourable clinical outcomes in a substantial proportion of patients. Prognosis varies markedly with stage at diagnosis. According to recent US population data, five-year relative survival rates are 92% for localised TNBC, 67% for regional disease, and 15% for metastatic disease, with an overall five-year relative survival of approximately 78% (American Cancer Society, 2026). These estimates were derived from population-based data for patients diagnosed with TNBC in the United States between 2015 and 2021. Surgical resection remains a principal treatment for localised tumours; however, metastatic TNBC remains difficult to treat because of its aggressive biology and propensity for rapid dissemination [14,15].

TNBC treatment is increasingly determined by disease stage and biomarker status [16,17]. In high-risk early-stage TNBC, treatment may include neoadjuvant chemotherapy combined with pembrolizumab, followed by surgery and adjuvant pembrolizumab. Patients with germline BRCA1/2 mutations may also benefit from adjuvant treatment with a PARP inhibitor [16,17]. For unresectable locally advanced or metastatic TNBC, treatment selection depends on factors such as PD-L1 expression, germline BRCA1/2 mutation status, HER2 expression, and previous therapy, and may include chemotherapy, immune-checkpoint inhibitors, PARP inhibitors, and antibody-drug conjugates [18,19,20].

Clinical management of TNBC is therefore complicated by its intrinsic molecular heterogeneity [21], the limited availability of effective targeted therapies [22], and a high risk of recurrence [23,24]. These difficulties are compounded by the limited number of reliable biomarkers available to predict or monitor treatment response, which can restrict therapeutic decision-making [25]. TNBC is also diagnosed at a younger age than several other breast cancer subtypes [24] and is associated with increased risks of metastasis and drug resistance [26,27,28]. Collectively, these challenges emphasise the need to identify new therapeutic targets and predictive biomarkers and to develop more personalised treatment strategies that improve patient outcomes.

This review adopts a clinically centred, problem-oriented framework to evaluate the major challenges associated with TNBC. Rather than considering clinical problems and therapeutic advances in isolation, it examines molecular heterogeneity, limited therapeutic targets, relapse, the shortage of validated biomarkers, younger age at diagnosis, metastasis, and drug resistance in relation to existing treatments and their limitations. This integrated approach aims to clarify how the principal clinical challenges of TNBC can inform the development and use of more precise and effective therapeutic strategies.

## 2. Primary Clinical Problems Associated with TNBC: Currently Available Therapies and Limitations

TNBC is a highly heterogeneous and biologically complex subtype of breast cancer, defined primarily by the absence of ER and PR expression and lack of HER2 positivity, together with variable molecular profiles and frequent dysregulation of oncogenic pathways [26]. This receptor-negative phenotype limits the utility of endocrine and HER2-targeted therapies and contributes to the distinct clinical behaviour of TNBC. Additional features associated with aggressive disease include a high incidence of visceral metastasis, early metastatic progression, rapid tumour growth, and relatively short durations of therapeutic response [29]. Consequently, patients with TNBC often have poorer outcomes than those with other breast cancer subtypes. Management is further complicated by molecular heterogeneity, the limited number of specific therapeutic targets, and the shortage of validated biomarkers for predicting treatment response [30]. Accordingly, only approximately 30–45% of patients achieve a pathological complete response (pCR), an outcome that is closely associated with survival [31]. Together, rapid proliferation, early dissemination, therapeutic resistance, and limited predictive biomarkers contribute to the aggressive clinical course of TNBC.

### 2.1. Molecular Heterogeneity and Subtypes

Gene-expression profiling has established TNBC as a molecularly heterogeneous disease comprising several biologically distinct subtypes, with important implications for diagnosis, prognosis, and therapeutic response. Lehmann et al. analysed gene-expression profiles from 587 TNBC cases and initially identified six subtypes: basal-like 1 (BL1), basal-like 2 (BL2), immunomodulatory (IM), mesenchymal (M), mesenchymal stem-like (MSL), and luminal androgen receptor (LAR) [32]. Subsequent studies revealed substantial overlap among these expression signatures and showed that the MSL and IM categories were strongly influenced by non-tumour components, including stromal and immune cells [33]. Lehmann and colleagues therefore refined the classification to four tumour-intrinsic subtypes-BL1, BL2, M, and LAR-after excluding MSL and IM [32]. Masuda et al. subsequently expanded the framework by including an unstable (UNS) group to account for tumours with undefined or mixed molecular characteristics [34]. Other classification systems have since been proposed to improve the clinical and diagnostic relevance of TNBC subtyping [21,35,36].

The refined Lehmann subtypes also differ in their responses to treatment. Among patients receiving neoadjuvant carboplatin plus docetaxel, BL1 tumours showed greater chemosensitivity, with a pathological complete response (pCR) rate of 41%, compared with 18% for BL2 and 29% for LAR tumours [32]. In a separate cohort treated with the same agents, pCR rates were again highest in BL1 tumours (65%), followed by BL2 (47.4%), M (36.4%), and LAR tumours (21.4%) [37]. Although survival differences among molecular subtypes have been investigated, their prognostic significance has not been consistently established [37].

Differences in key oncogenes, tumour-suppressor genes, and the immune microenvironment contribute to variation in therapeutic response among TNBC subtypes [38]. This biological diversity can alter sensitivity to chemotherapy and targeted agents and remains a major barrier to the development of broadly effective, U.S. FDA-approved targeted treatments [32,38]. To improve patient stratification and personalise therapy, increasing emphasis is being placed on integrated profiling of transcriptomic and epigenomic features together with characterisation of the tumour immune microenvironment [38].

**Figure 1:**
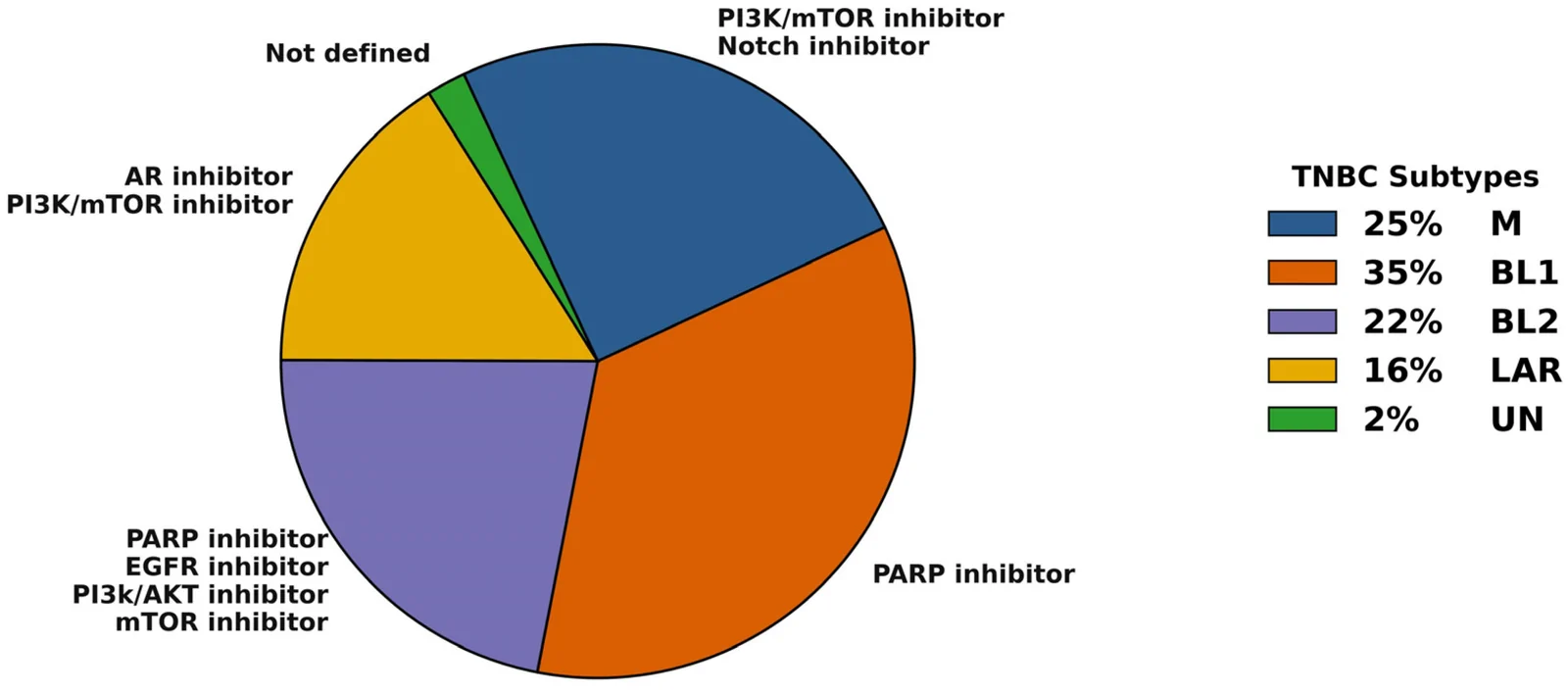
Molecular subtypes of TNBC, their percentage distribution (adapted from [32]), and potential therapeutic targets associated with individual subtypes (adapted from [43]). UN represents the unclassified category.

Figure 1 summarises the principal molecular subtypes of TNBC, their reported distribution, and potential therapeutic targets. A precision-medicine approach seeks to align treatment with the molecular and immunological features of individual tumours. Transcriptomic analyses, for example, can identify gene-expression patterns associated with therapeutic sensitivity or resistance and have revealed biomarkers linked to chemotherapy and immunotherapy response in TNBC [38,39]. Epigenetic alterations, including DNA methylation and histone modifications, can also influence tumour gene expression and may provide predictive information regarding treatment efficacy [40]. Integrating transcriptomic, epigenetic, and immune-cell data may therefore provide a more comprehensive view of TNBC biology and support the development of personalised immunotherapeutic and targeted strategies [32,36,41,42].

### 2.2. Limited Options for Targeted Therapy

Targeted therapy in breast cancer is directed towards molecular dependencies that drive tumour growth and progression, most prominently ER and HER2 [44]. Most breast cancers are ER-positive, and approximately half of these also express PR, whereas only a small proportion (~2%) are ER-positive but PR-negative [45]. Because many breast cancers express ER without HER2 overexpression, endocrine and HER2-directed therapies have transformed the management of receptor-defined disease [46]. TNBC, by definition, lacks ER and PR expression and does not meet criteria for HER2-positive disease; the HER2-negative category includes HER2-zero and HER2-low tumours [45]. This receptor profile, together with marked molecular heterogeneity, limits therapeutic options and contributes to poorer outcomes despite advances in TNBC biology [42]. Although chemotherapy remains central to treatment, clinically established options now also include biomarker- and setting-specific use of immune-checkpoint inhibitors, PARP inhibitors, and antibody-drug conjugates [16,19,20]. Numerous additional pathways and targets remain under investigation, including PI3K/AKT/mTOR [47], EGFR [48], Notch [49], PARP [50], antibody-drug conjugates [51], CDKs [52], androgen receptor (AR) [53], FGFR [54], VEGFR [55], TGF-β [56], STAT3 [57], epigenetic regulators [58], immunotherapeutic targets [43], combination regimens [59], and regulated cell-death pathways [42] (Table 1).

Immunotherapeutic strategies, including immune-checkpoint blockade, cancer vaccines, and adoptive cell transfer, have been evaluated in clinical studies of TNBC and have produced encouraging results in selected settings [43,60]. Nevertheless, drug resistance, treatment-related toxicity, and limited benefit in unselected patient populations remain important challenges [61]. Advances in molecular subtyping, targeted-therapy development, immunotherapy, and histopathological characterisation are progressively refining precision-medicine approaches to TNBC [62]. The dysregulation of multiple oncogenic signalling pathways during disease progression also provides a basis for identifying diagnostic and prognostic biomarkers and for developing new therapeutic strategies. Although several pathway-directed agents have shown promise in preclinical models, their successful integration into clinical practice will require rigorous validation and appropriate patient selection [43].

**Table 1:** Established and investigational therapeutic approaches in TNBC: molecular targets, representative agents, clinical settings, and key limitations.

Therapeutic Class	Molecular Target	Representative Drugs/Inhibitors	Clinical Status/Setting	Key Limitations	References
Chemotherapy	DNA repair	Doxorubicin, Epirubicin	Established (in breast cancer)	Cardiotoxicity, bone marrow suppression, drug resistance, limited efficacy in metastatic disease, risk of secondary malignancies	[63]
	Microtubule stabilization	Paclitaxel, Docetaxel, Cabazitaxel	Paclitaxel, Docetaxel-Established (in breast cancer); Cabazitaxel-Investigational (in TNBC)	Peripheral neuropathy, myelosuppression, hypersensitivity reactions, drug resistance	[63,64,65]
	DNA crosslinking/platinum agent	Carboplatin	Established (in TNBC)	Toxicity, onycholysis, epiphora, acquired resistance	[66,67]
Targeted Therapy	PI3K/AKT/mTOR pathway	Alpelisib, Everolimus, Temsirolimus, Buparlisib, Gedatolisib, Capivasertib, Inavolisib, others	Investigational (in TNBC)	Minimal efficacy, off-target toxicity (e.g., hyperglycemia, rash), resistance due to pathway mutations, need for subtype-specific therapies	[43,68,69,70]
	EGFR	Cetuximab, Erlotinib, Gefitinib, Lapatinib, Afatinib, Panitumumab	Investigational (in TNBC)	Low susceptibility of TNBC cells to EGFR inhibition, lack of EGFR dependence	[43,63,71,72]
	Notch signalling	RO4929097, AL101	Investigational (in TNBC)	Gastrointestinal toxicity, dose-dependent side effects	[63,73]
	PARP	Olaparib, Talazoparib, Rucaparib, Niraparib, Veliparib	Olaparib, Talazoparib-FDA-approved (for eligible gBRCA-mutated HER2-negative breast cancer); Rucaparib, Niraparib, Veliparib-Investigational (in TNBC)	Efficacy restricted to BRCA1/2-mutant TNBC; resistance via BRCA reversion mutations	[63,74,75]
	CDK4/6	Palbociclib, Abemaciclib, Trilaciclib	Palbociclib, Abemaciclib-FDA-approved (for HR+/HER2− breast cancer); Trilaciclib-Investigational (in TNBC)	Toxicity (e.g., fatigue, visual disturbances), lack of pharmacokinetic studies, poor combination regimen definition	[43,76,77]
	Androgen Receptor (AR)	Bicalutamide, Enzalutamide, GTx-024	Investigational (in TNBC)	AR expression in 12–36% of TNBC cases limits applicability; response is molecular subtype-dependent	[43,63]
	FGFR	AZD4547, Erdafitinib, Futibatinib	Investigational (in TNBC)	Lack of predictive biomarkers, drug resistance, unclear patient selection	[78]
	VEGF/VEGFR	Bevacizumab, Ramucirumab, Sorafenib, Apatinib	Bevacizumab- Formerly approved (U.S. metastatic breast cancer indication withdrawn in 2011); ramucirumab, sorafenib and apatinib- Investigational (in TNBC)	Limited clinical efficacy despite promising preclinical results	[43,72,79,80,81]
	TGF-β	Bintrafusp Alfa (M7824), Galunisertib	Investigational (in TNBC)	Limited understanding of effects on cancer stem cell pathways; optimization of combination therapies needed	[43,82]
	STAT3	Carfilzomib, Bazedoxifene, Nintedanib, Arctigenin, Niclosamide, Metformin, others	Investigational (in TNBC)	Limited direct binding evidence to STAT3; preclinical data only	[83,84]
	Epigenetic modulation	Belinostat, Chidamide, Entinostat (HDAC); Decitabine (DNA methylation)	Investigational (in TNBC)	Off-target toxicity; limited efficacy as monotherapy in solid tumours; need for selective modulators	[43,58,85,86]
	JAK2	Ruxolitinib	Investigational (in TNBC)	Safety concerns; not approved for TNBC by FDA	[83,87]
	CHK1	AZD7762, PF477736, SCH900776, Prexasertib	Investigational (in TNBC)	Unpredictable cardiac effects; variable efficacy across patient groups	[88,89]
	Src	Dasatinib	Investigational (in TNBC)	Innate resistance; unsatisfactory clinical trial results for single-agent use	[76,90,91]
Immunotherapy	Immune checkpoints (PD-1, PD-L1, CTLA-4)	Pembrolizumab, Nivolumab, Atezolizumab, Durvalumab, Ipilimumab	Pembrolizumab-FDA-approved (in defined TNBC settings); Atezolizumab-Formerly approved (U.S. TNBC indication withdrawn); Nivolumab, Durvalumab, Ipilimumab-Investigational (in TNBC)	Low microsatellite instability in TNBC limits immunotherapy responsiveness	[91,92,93,94,95,96]
	CAR-T Cells	cMet CAR-T, ROR1-CAR-T, CART-TnMUC1	Investigational (in TNBC)	Limited to pediatric or specific adult cases; risk of cross-reactivity and severe toxicity	[97]
	Antibody–Drug Conjugates (ADC)	Sacituzumab Govitecan, Datopotamab deruxtecan, T-DXd, TH1902, Vobramitamab Duocarmazine	Sacituzumab govitecan, Datopotamab deruxtecan-FDA-approved (in defined TNBC settings); T-DXd-FDA-approved (in defined HER2-expressing breast-cancer settings); TH1902, Vobramitamab duocarmazine-Investigational (in TNBC)	Resistance mechanisms, absence of robust predictive biomarkers, need for rational combinations	[20,97,98,99]

### 2.3. High Risk of Relapse

TNBC has a high risk of recurrence, creating a major challenge for long-term disease control [100]. Clinical factors associated with recurrence include larger tumour size, lymph-node involvement, and the need for neoadjuvant therapy [100,101,102]. Tumour size is among the most consistent predictors of relapse, with larger tumours carrying increased risk even in the absence of nodal metastasis [101]. Recent studies have also implicated quiescent cancer cells (QCCs), a subpopulation characterised by transcriptional programmes associated with chemoresistance, hypoxia, and altered glycolysis, in TNBC recurrence [103]. Cancer stem cells, including populations that can remain arrested in the G0 phase, may contribute to tumour dormancy and resistance to therapy [103].

The first three years after diagnosis represent a particularly high-risk period for recurrence in TNBC. Recurrence rates of approximately 30–40% have been reported in operable disease and may approach 50% among patients who do not achieve a pathological complete response (pCR) after neoadjuvant systemic therapy [23,102,104]. Despite advances in systemic treatment, median overall survival for metastatic TNBC remains below two years [104]. Although approximately 60% of patients with stage I–III TNBC achieve long-term disease-free survival, around 40% experience recurrence despite standard treatment [105]. Recurrence also tends to occur earlier than in non-TNBC subtypes, often within 19–40 months [106,107]. Organ-specific analyses have reported the liver and central nervous system (CNS) as important sites of relapse, accounting for approximately 59.2% and 30.6% of recurrences, respectively, in the cited cohort [100].

Because failure to achieve pCR is strongly associated with recurrence, residual disease after neoadjuvant therapy is an important determinant of subsequent management in TNBC [108]. Post-neoadjuvant treatment can therefore be individualised according to residual disease, previous therapy, and molecular biomarkers. Adjuvant capecitabine has been associated with improved overall and disease-free survival in patients with HER2-negative breast cancer who have residual invasive disease after neoadjuvant chemotherapy, with a notable benefit in the TNBC subgroup [109]. In patients treated with the KEYNOTE-522 regimen, pembrolizumab is continued after surgery, and clinical benefit has also been observed in many patients with residual disease [110,111]. For patients with germline BRCA1/2 mutations and high-risk HER2-negative early breast cancer, adjuvant olaparib provides an additional biomarker-guided option [17]. However, the optimal combination and sequencing of capecitabine, pembrolizumab, and olaparib in patients eligible for more than one of these therapies remain uncertain.

### 2.4. Limited Biomarkers for Predicting and Monitoring Treatment Outcome

The National Cancer Institute (NCI) defines a biomarker as a biological molecule found in tissue, blood, or other body fluids that can indicate a normal or abnormal process, condition, or disease. In clinical oncology, biomarkers may provide prognostic information or predict treatment response (FDA-NIH Biomarker Working Group, 2016). Numerous biomarkers have been investigated in TNBC, although the strength of clinical evidence varies considerably. PD-L1 expression is an established predictive biomarker in advanced TNBC, where a combined positive score (CPS) ≥10 identifies patients who may benefit from pembrolizumab-based treatment, whereas germline BRCA1/2 mutations inform the use of PARP inhibitors [19,112]. Stromal tumour-infiltrating lymphocytes (TILs) are a useful prognostic factor in early-stage TNBC, with higher TIL levels associated with improved survival [113] and higher pCR rates following neoadjuvant chemotherapy [114]. Liquid-biopsy approaches may provide additional prognostic information: elevated circulating tumour-cell (CTC) levels are associated with poorer outcomes in metastatic breast cancer, whereas detectable circulating tumour DNA (ctDNA) after treatment can indicate molecular residual disease and an increased risk of recurrence [115,116]. Studies of ctDNA monitoring suggest that molecular residual disease may be detectable before clinical relapse in high-risk early TNBC, although routine clinical implementation remains under investigation [117]. Other alterations-including PI3K/AKT-pathway changes, androgen receptor expression, EGFR, DNA methylation patterns, and genomic signatures-are being explored for prognostic or therapeutic value but currently have more limited roles in routine TNBC management. Next-generation sequencing (NGS)-based profiling has identified potentially relevant alterations in genes such as TP53, KDR, PIK3CA, ATM, and AKT1 that may inform target discovery and precision-treatment strategies [118,119,120,121,122].

### 2.5. Younger Age at Diagnosis

Breast cancer is an important cause of cancer-related mortality in women younger than 40 years [123,124,125], and nearly 7% of women with breast cancer are diagnosed before this age [126]. TNBC, in particular, is more frequently diagnosed at a younger age than several other breast cancer subtypes [24,102,126]. Hereditary predisposition may partly contribute to this pattern. In a cohort of 1824 patients with TNBC, pathogenic germline variants were identified in 14.6%, including BRCA1 (8.5%), BRCA2 (2.7%), PALB2 (1.2%), and less frequent variants in BARD1, RAD51D, RAD51C, and BRIP1 [127]. Another study identified BARD1, BRCA1, BRCA2, PALB2, and RAD51D as genes associated with increased TNBC risk [128]. Several reports suggest that younger patients with TNBC may have less favourable outcomes than older patients [126], although the prognostic effect of age appears to depend on the comparison group and may be influenced by molecular subtype [129]. Age-related differences in biomarkers, including proliferative markers, HER2, and endocrine-receptor expression, have also been reported [126]. However, the independent prognostic significance of early-onset TNBC remains uncertain [129]. Some studies have examined outcomes by molecular subtype [130,131,132], with findings varying according to the age thresholds used: younger patients showed better breast cancer-specific survival and overall survival than groups defined as >60 years, but worse outcomes than groups defined as ≥40 years [129]. Pooled analyses also suggest a higher risk of recurrence in early-onset disease [129]. Even after accounting for stage, grade, histology, treatment, and surgery, younger age at diagnosis has historically been associated with poorer survival and higher risks of local and contralateral recurrence [126,133]. These uncertainties support the need for clinical studies designed to clarify the biological and therapeutic implications of early-onset TNBC and to optimise management for younger patients.

### 2.6. High Risk of Metastasis

Despite surgery and adjuvant therapy, a substantial proportion of patients with TNBC initially confined to the breast and regional lymph nodes develop distant recurrence within five years, with a particular propensity for visceral and central nervous system metastases [134]. Among breast cancer subtypes, TNBC has a high frequency of distant metastasis and comparatively poor overall survival [135]. Different TNBC subtypes may show distinct patterns of organ tropism, with the brain and lungs representing important metastatic sites [27]. Brain metastasis is a particularly serious complication and is associated with poor prognosis [136]. In a cohort of 433 patients with metastatic TNBC, 29% developed brain metastases, and median overall survival after brain-metastasis diagnosis was 7.3 months [137]. Treatment is complicated by the blood–brain barrier, which can restrict the intracranial penetration of systemic therapies [136]. Local approaches-including stereotactic radiosurgery, whole-brain radiotherapy, and surgical resection-remain important depending on the number, size, and location of lesions and the patient’s clinical condition [136]. Although systemic treatment is evolving, TNBC brain metastasis remains a major unmet clinical need, underscoring the importance of therapies with improved intracranial activity [136].

Aberrant activation of oncogenic signalling pathways also contributes to TNBC invasion and metastasis. PI3K/AKT/mTOR signalling can promote epithelial-to-mesenchymal transition (EMT), migration, and invasive behaviour, whereas PTEN alterations and mTOR activation have been associated with metastatic phenotypes [138,139]. Wnt/β-catenin signalling can likewise enhance migration and invasion, and pathway activation has been linked to lung and brain metastases [140]. STAT3 signalling further contributes to invasion and dissemination by regulating transcriptional programmes associated with EMT and metastatic behaviour [141,142].

The high metastatic potential of TNBC contributes substantially to its mortality [14]. Despite therapeutic advances, approximately 25–40% of patients with breast cancer ultimately develop metastatic disease, which remains largely incurable [143]. Systemic treatment for metastatic TNBC has expanded beyond conventional chemotherapy to include immune-checkpoint inhibitors, PARP inhibitors, and antibody-drug conjugates (ADCs). Pembrolizumab-based therapy is a standard first-line option for patients with PD-L1-positive (CPS ≥ 10) unresectable locally advanced or metastatic TNBC [112]. ADCs have also moved into earlier treatment lines, further broadening systemic options for advanced disease [144]. Combinations of immunotherapy and chemotherapy, including atezolizumab- or pembrolizumab-based regimens, have shown benefit in selected patients with PD-L1-positive metastatic TNBC [145], although not all combinations have been successful; for example, atezolizumab plus paclitaxel did not improve outcomes in the cited trial [145]. Considerable effort is therefore focused on improving immunotherapy responsiveness in PD-L1-negative disease and on overcoming resistance after prior immune-checkpoint inhibition [104]. PARP inhibitors have produced higher response rates and longer progression-free survival than chemotherapy in patients with germline BRCA1/2 mutations treated in the first- or second-line setting [19,146]. Preclinical studies have also explored combinations such as olaparib with orally administered oligo-fucoidan for the prevention of postsurgical recurrence and metastasis [147,148,149]. Another rapidly developing strategy is the use of novel ADCs, which couple tumour-targeting antibodies to potent cytotoxic payloads [104,150]. However, resistance remains a major limitation. For sacituzumab govitecan, resistance may involve alterations in the target or cytotoxic payload, reduced antigen expression, impaired internalisation or lysosomal processing, drug efflux, and changes in pathways governing payload sensitivity [150,151]. Agents such as trastuzumab duocarmazine and datopotamab deruxtecan illustrate the expanding ADC landscape, although their distinct toxicities require careful clinical consideration [20,152,153]. A clearer understanding of resistance to both immunotherapy and ADCs will be essential for improving the durability of responses in metastatic TNBC.

### 2.7. Drug Resistance

Drug resistance remains a major obstacle in TNBC management and is an important contributor to treatment failure and cancer-related mortality [154]. Resistance can be broadly classified as intrinsic, in which reduced sensitivity is present before treatment, or acquired, in which resistance emerges during therapy [155]. Multiple mechanisms contribute to resistance in breast cancer, including increased drug efflux, enhanced DNA-repair capacity, epigenetic changes, intertumoural heterogeneity, and epithelial-to-mesenchymal transition (EMT) [155]. Overexpression of ATP-binding cassette (ABC) transporters such as ABCB1, ABCC1, and ABCG2 can promote multidrug resistance by exporting chemotherapeutic agents from cancer cells and thereby reducing intracellular drug concentrations [155]. Other transporters, including ABCC3 and ABCC6, have also been implicated in altered intracellular drug distribution or sequestration [155]. Beyond drug efflux, oncogenic signalling can promote therapeutic resistance. Activating alterations in PIK3CA can stimulate PI3K/AKT/mTOR signalling, reduce apoptosis induced by agents such as epirubicin and cisplatin, and promote drug resistance, whereas pathway inhibition may restore sensitivity [156]. Resistance can also emerge to commonly used cytotoxic agents. Cisplatin resistance has been linked to altered DNA-repair pathways and reduced drug uptake [157]; gemcitabine resistance has been associated with regulatory microRNAs, including miR-873 [158]; methotrexate resistance has been associated with differential expression of multiple proteins [159]; and resistance to 5-fluorouracil has been linked to the anti-apoptotic protein BCL2A1 [160]. Defining the molecular basis of these resistance mechanisms is therefore important for developing more durable treatment strategies [161].

These major clinical challenges of TNBC are interrelated and highlight the limitations of current treatment approaches. Table 2 summarises these challenges alongside existing management strategies, their limitations, relevant biomarkers, and emerging therapeutic approaches, with their corresponding levels of evidence.

## 3. Emerging Therapeutic Perspectives in TNBC

The major clinical challenges in TNBC are closely interconnected and collectively limit the effectiveness of a uniform treatment strategy. Molecular heterogeneity and the restricted number of actionable targets support the use of biomarker-guided approaches, whereas relapse, metastasis, and drug resistance require strategies that improve response durability and overcome treatment failure. The following sections discuss emerging approaches aimed at refining patient selection, enhancing therapeutic response, circumventing resistance, and directly targeting molecular mechanisms that sustain TNBC.

### 3.1. Biomarker-Guided Precision Therapy

Biomarker-guided precision therapy is increasingly important in TNBC because of the disease’s molecular heterogeneity, diverse immune landscapes, and variable treatment responses [30,163]. Established predictive biomarkers, such as PD-L1 expression and germline BRCA1/2 mutations, can help stratify patients for immunotherapy and PARP inhibitors, respectively, while homologous-recombination deficiency and androgen-receptor status remain investigational [163,164]. Trop-2 serves as a therapeutic target for antibody-drug conjugates, although its expression is not a clinically validated predictive biomarker for patient selection [20,165]. Key biomarker-treatment relationships are summarised in Table 3. By matching therapy to tumour biology, biomarker-guided treatment may reduce unnecessary toxicity, improve treatment selection, and support more individualised management rather than a uniform approach for all patients with TNBC [30].

Clinical and molecular factors influence not only treatment selection but also therapeutic sequencing, particularly in advanced TNBC, where patients may receive multiple lines of systemic therapy. In previously untreated PD-L1-positive (CPS ≥ 10) advanced TNBC, pembrolizumab-based therapy can provide substantial clinical benefit [112,144]. After progression, subsequent treatment depends on previous exposure, biomarker status, and remaining options and may include PARP inhibitors for patients with germline BRCA1/2 mutations or ADCs in appropriate settings [19,20]. Treatment sequencing should therefore be individualised according to both tumour biology and prior therapy.

### 3.2. Immunotherapy

Immunotherapy has substantially altered the treatment landscape of TNBC, primarily through immune-checkpoint inhibitors targeting the PD-1/PD-L1 axis [171]. In high-risk early-stage TNBC, neoadjuvant pembrolizumab plus chemotherapy followed by adjuvant pembrolizumab has improved pathological complete response, event-free survival, and overall survival compared with chemotherapy alone [16]. In advanced disease, pembrolizumab-based chemoimmunotherapy provides benefit in selected patients with PD-L1-positive tumours [112], underscoring the importance of biomarker-guided patient selection. Nevertheless, primary and acquired resistance, immune-related adverse events, and limited benefit in some biomarker-negative tumours remain major limitations [172]. Management after progression on immune-checkpoint inhibitors is also challenging because no single post-immunotherapy treatment sequence has been established for TNBC [173]. Subsequent therapy is generally guided by previous treatment exposure and available options, including chemotherapy, ADCs, and PARP inhibitors [19,20]. Beyond immune-checkpoint blockade, emerging approaches include personalised mRNA cancer vaccines and CAR-based cellular therapies. A personalised neoantigen mRNA vaccine has generated tumour-specific T-cell responses in patients with TNBC, supporting further clinical evaluation [174]. CAR-NK and CAR-macrophage approaches are also under investigation; however, current evidence is largely preclinical, and tumour-antigen heterogeneity and the immunosuppressive tumour microenvironment remain important barriers [175].

### 3.3. Combination Therapy

Combination therapy is increasingly important in TNBC because relapse and treatment failure often arise through multiple, interacting mechanisms, including immune escape, altered DNA-repair pathways, tumour heterogeneity, and activation of compensatory survival signalling [176]. Examples include chemotherapy combined with immune-checkpoint inhibitors, PARP inhibitors combined with platinum agents or immunotherapy, and inhibitors of DNA-damage-response proteins such as ATR, CHK1, or WEE1 in selected molecular contexts [177,178,179]. Antibody-drug conjugates (ADCs) have also become central to combination strategies because they can deliver potent cytotoxic payloads selectively to tumour cells and may complement immunotherapy or other targeted agents [180]. The clinical relevance of ADC-based treatment is illustrated by Trop-2-directed agents such as sacituzumab govitecan and datopotamab deruxtecan, which have entered defined locally advanced or metastatic TNBC settings [20,99]. More recently, sacituzumab govitecan combined with pembrolizumab received FDA approval in 2026 for first-line treatment of unresectable locally advanced or metastatic TNBC [144].

### 3.4. Targeted Protein Degradation

Targeted protein degradation is an emerging therapeutic strategy that removes disease-associated proteins through the ubiquitin-proteasome system rather than merely inhibiting their activity [181]. Proteolysis-targeting chimeras (PROTACs) are a major class of targeted protein degraders [181]. These heterobifunctional molecules contain a ligand for the protein of interest, a linker, and a ligand that recruits an E3 ubiquitin ligase, thereby bringing the target protein into proximity with the ubiquitination machinery and promoting proteasomal degradation [182]. Several TNBC-associated proteins, including BRD4, EGFR, AKT, CDKs, and the androgen receptor, are being investigated as PROTAC targets [183,184]. A BRD4-directed PROTAC, compound 6b, has been reported to induce CRBN-mediated BRD4 degradation, suppress KLF5-associated oncogenic signalling, inhibit basal-like breast cancer-cell growth, and reduce tumour proliferation in HCC1806 xenografts [185]. EGFR degradation may provide an alternative to conventional receptor inhibition by reducing total receptor abundance, although this strategy remains largely preclinical in TNBC [162]. AKT degraders, including MS21 and newer AKT-directed PROTACs, have also shown suppression of AKT signalling and anticancer activity [186]. Similarly, PROTACs targeting CDK1, CDK4/6, CDK9, and CDK12/13 may interfere with cell-cycle progression, transcriptional regulation, and DNA-damage-repair pathways [162], whereas AR-directed degraders may be particularly relevant to AR-positive or luminal androgen-receptor TNBC [162]. Recent studies have broadened the range of candidate targets. The CRBN-recruiting PROTAC YZ-836P induced PRMT5 degradation, reduced downstream KLF5, promoted cell-cycle arrest and apoptosis, and suppressed TNBC growth in organoid and xenograft models [187]. An orally available compound, PROTAC 29, produced selective CDK9 degradation, reduced c-Myc and MCL-1, and inhibited MDA-MB-231 xenograft growth after oral administration [188]. The NAMPT-targeting PROTAC A13 has also been reported to inhibit TNBC proliferation, migration, and invasion, promote M1-like tumour-associated macrophage polarisation, and suppress primary tumour growth and lung metastasis in an orthotopic model [189]. Thus, targeted degradation may expand the druggable space in TNBC by enabling the removal of oncogenic proteins that are difficult to inhibit conventionally. However, most TNBC-directed PROTACs remain at the preclinical stage, and important challenges include bioavailability, tissue delivery, E3-ligase selection, selectivity, resistance, toxicity, and biomarker development [162]. The FDA approval of vepdegestrant for ER-positive/HER2-negative, ESR1-mutated advanced or metastatic breast cancer provides clinical validation of targeted protein degradation in breast cancer, although this agent is not approved for TNBC [190]. This development supports continued evaluation of TNBC-directed protein degraders.

## 4. Conclusions

TNBC remains a clinically challenging breast cancer subtype because aggressive behaviour, molecular heterogeneity, early relapse, metastatic potential, drug resistance, and the limited availability of validated predictive biomarkers restrict the effectiveness of current therapies. There is therefore a pressing need for improved biomarkers and personalised treatment strategies. Because TNBC comprises biologically distinct subtypes, only selected patient groups are likely to benefit from any single targeted agent. Defining the molecular mechanisms that drive individual subtypes and identifying robust subtype-specific biomarkers will be essential for rational treatment selection and the development of more effective combination strategies. Progress will also depend on closer integration of basic, translational, and clinical research. Ultimately, improving outcomes in TNBC will require the conversion of mechanistic insights into biomarker-informed therapies that provide durable disease control while preserving quality of life.

## Figures and Tables

**Table 2:** Integrating Clinical Challenges, Therapeutic Limitations, and Emerging Strategies in TNBC.

Clinical Challenge	Current Management	Major Limitations	Relevant Biomarkers/Molecular Features	Emerging Solutions	Evidence Level	References
Molecular Heterogeneity and Subtypes	Chemotherapy and selected targeted therapies	Different subtypes respond differently to treatment	BL1, BL2, M, LAR	Molecular profiling and personalised therapy	Clinical and translational; emerging approaches investigational	[32,37,38]
Limited Targeted Therapy	Chemotherapy, immunotherapy, PARP inhibitors and ADCs	Few actionable targets; toxicity and resistance	PD-L1, germline BRCA1/2	PI3K/AKT/mTOR inhibitors, AR-targeted therapy and PROTACs	Phase III for established therapies; preclinical/early clinical for emerging approaches	[16,19,20,47,53,162]
High Risk of Relapse	Neoadjuvant therapy; adjuvant pembrolizumab, capecitabine or olaparib in eligible patients	Residual disease and early recurrence	pCR, residual cancer burden, ctDNA	ctDNA-guided monitoring and risk-adapted therapy	Phase III for established treatments; investigational for ctDNA-guided treatment	[17,103,109,110,111]
Limited Biomarkers	Treatment selection using validated biomarkers	Few reliable markers for predicting response and recurrence	PD-L1, BRCA1/2, TILs, ctDNA	Liquid biopsy and multi-omics profiling	Phase III for treatment-selection markers; observational/investigational for other markers	[19,112,113,114,116,117]
Younger Age at Diagnosis	Standard treatment with genetic counselling and age-specific care	Hereditary risk and uncertain age-related prognosis	BRCA1/2, PALB2, BARD1	Genetic risk assessment and age-specific management	Genetic/observational studies; consensus guidelines	[127,128,129,130]
High Risk of Metastasis	Chemotherapy, immunotherapy, PARP inhibitors, ADCs and local CNS treatment	Early distant spread and poor CNS drug penetration	PD-L1, germline BRCA1/2	CNS-active therapies and novel treatment combinations	Phase III for systemic therapies; observational for CNS outcomes; emerging approaches investigational	[19,112,134,136,137]
Drug Resistance	Chemotherapy and subsequent-line systemic therapies	Drug efflux, enhanced DNA repair and activation of survival pathways	ABC transporters, PIK3CA, DNA-repair alterations	Combination therapy and targeted protein degradation	Clinical evidence for existing treatments; mainly preclinical/translational for resistance-directed approaches	[150,155,156,157,161,162]

**Table 3:** Biomarker-guided therapeutic options in TNBC.

Biomarker Status	Treatment Direction	Clinical Status	Disease Setting	References
PD-L1 CPS ≥ 10	Pembrolizumab + chemotherapy; pembrolizumab + sacituzumab govitecan in eligible unresectable locally advanced or metastatic TNBC	FDA-approved	Advanced/metastatic TNBC	[112,144]
High-risk early TNBC	Neoadjuvant pembrolizumab + chemotherapy followed by adjuvant pembrolizumab	FDA-approved	Early-stage TNBC	[16]
gBRCA1/2 mutation	Olaparib or talazoparib; adjuvant olaparib in selected high-risk HER2-negative early breast cancer	FDA-approved	Early-stage and advanced/metastatic TNBC	[17,19,146]
HRD/DNA repair defect	Platinum sensitivity; PARP inhibitor or DDR-targeted combinations under evaluation	Investigational	Clinical trials	[50]
HER2-low TNBC	Trastuzumab deruxtecan in appropriate unresectable or metastatic HER2-low breast cancer setting	FDA-approved	Advanced/metastatic TNBC	[166]
AR-positive TNBC	AR-blockade or AR-degrader approaches	Investigational	Clinical trials	[167]
MSI-H/dMMR or TMB-high	Pembrolizumab	FDA-approved (tumour-agnostic indication)	Advanced/metastatic solid tumours	[16,168,169]
NTRK fusion	Larotrectinib or entrectinib	FDA-approved (tumour-agnostic indication)	Advanced/metastatic solid tumours	[170]

## Data Availability

Not applicable. No new data were created or analyzed in this study. Data sharing is not applicable to this article.
